# Inductive generalization with familiar categories: developmental changes in children's reliance on perceptual similarity and kind information

**DOI:** 10.3389/fpsyg.2015.00897

**Published:** 2015-07-07

**Authors:** Karrie E. Godwin, Anna V. Fisher

**Affiliations:** Department of Psychology, Carnegie Mellon UniversityPittsburgh, PA, USA

**Keywords:** categorization, induction, reasoning, children, cognitive development

## Abstract

Inductive generalization is ubiquitous in human cognition; however, the factors underpinning this ability early in development remain contested. The present study was designed to (1) test the predictions of the naïve theory and a similarity-based account and (2) examine the mechanism by which labels promote induction. In Experiment 1, 3- to 5-year-old children made inferences about highly familiar categories. The results were not fully consistent with either theoretical account. In contrast to the predictions of the naïve theory approach, the youngest children in the study did not ignore perceptually compelling lures in favor of category-match items; in contrast to the predictions of the similarity-based account, no group of participants favored perceptually compelling lures in the presence of dissimilar-looking category-match items. In Experiment 2 we investigated the mechanisms by which labels promote induction by examining the influence of different label types, namely category labels (e.g., the target and category-match both labeled as *bird*) and descriptor labels (e.g., the target and the perceptual lure both labeled as *brown*) on induction performance. In contrast to the predictions of the naïve theory approach, descriptor labels but not category labels affected induction in 3-year-old children. Consistent with the predictions of the similarity-based account, descriptor labels affected the performance of children in all age groups included in the study. The implications of these findings for the developmental account of induction are discussed.

## Introduction

Inductive reasoning involves making generalizations from instances. It is a powerful and effective tool for generating new knowledge. Consider this example: when told a novel fact about alligators (e.g., “alligator embryos lack sex chromosomes”) most adults correctly conclude that crocodile embryos also lack sex chromosomes. Thus, making an inductive inference on the basis of what is known *creates* new knowledge. This mode of inference is not guaranteed to generate correct knowledge (one might incorrectly overgeneralize a fact about alligator embryos to all oviparous animals). Nevertheless, the ability to make such inferences is a hallmark of mature cognition.

In the absence of perceptual information to guide inferences, adults have been hypothesized to make inferences on the basis of their knowledge about object kind: objects that belong to the same or related categories are likely to have many properties in common (e.g., Rips, 1975; Osherson et al., 1990; Kemp and Tenenbaum, 2008; Hayes et al., 2010; Murphy and Ross, 2010). In other words, adults are said to use category-based induction to generalize from the known to the unknown. Despite general agreement that category-based induction is a ubiquitous component of mature cognition (although see Sloman, 1993), there is little agreement regarding the developmental origins of this ability. In particular, it remains unclear whether young children utilize object kind information in the course of induction and what role linguistic labels play in this process. In recent years, the debate on the development of category-based induction has been dominated by two alternative perspectives: a naïve theory account (Gelman and Markman, 1986; Markman, 1990) and a similarity-based account (Sloutsky and Fisher, 2004, 2012). Two fundamental differences between these accounts are: (1) whether category-based induction *emerges* gradually in the course of development or whether children are *initially predisposed* to rely on object kind information in the course of induction and (2) whether linguistic labels contribute to inductive inference by providing information about object kind or by increasing featural overlap among presented entities. Below we provide a brief overview of each account, focusing on these two major distinctions.

According to the naïve theory approach, from very early in development people first identify the *category membership* of items under consideration and then generalize a known property to items of the same kind: “by 2 ½ years, children expect categories to promote rich inductive inferences… and they can overlook conflicting perceptual appearances in doing so” (Gelman and Coley, 1990, p. 802). Furthermore, it has been suggested that the ability to make category-based inferences is not a product of development and learning. Instead, children are “initially biased” to recognize that *labels denote categories* and make inferences on the basis of shared labels and hence shared category membership (Gelman and Markman, 1986, p. 207), an idea that has been highly influential in the literature (e.g., Keil, 1989; Gelman and Coley, 1990; Booth and Waxman, 2002; Kalish, 2006; Jaswal and Markman, 2007). Thus, under the naïve theory account, from a very early age children are expected to make category-based inductions even if perceptual information is in conflict with category membership information. As a result, one would expect even young children to perform relatively well on simple induction tasks when category membership information is readily available. Additionally, any observed improvement in performance with age is thought to be due to a reduction in statistical noise rather than changes in the mechanisms of induction.

In contrast to the naïve theory approach, Sloutsky and Fisher (2004) proposed a similarity-based account called SINC (for Similarity, Induction, Naming, and Categorization). According to SINC, children make inferences on the basis of the overall similarity of presented entities computed over all perceived object features. Within this approach, labels are considered to be object features (rather than category markers) that contribute to the overall perceived similarity. Therefore, according to SINC an inference can be label-based without necessarily being category-based. Several findings suggest that children rely primarily on overall perceptual similarity of objects (but not category membership information) to make inferences well beyond the preschool years, possibly until 7–9 years of age (e.g., Fisher and Sloutsky, 2005; Sloutsky et al., 2007; Badger and Shapiro, 2012; Sloutsky and Fisher, 2012). In other words, in contrast to the early competence notion advocated by the proponents of the naïve theory account, proponents of SINC suggest that category-based induction is not a developmental default, but instead category-based induction follows a protracted developmental course.

Perhaps the strongest evidence for the naïve theory account comes from the seminal study by Gelman and Markman (1986). In this study researchers asked preschool-age children and college students to make inferences about natural kind objects when perceptual information was ambiguous or conflicted with category membership (cf. Sloutsky and Fisher, 2004). Labels were used to communicate category information; for instance, participants were asked whether a target item (e.g., a brown *squirrel*) shared a non-obvious property with the test item that was designed to look similar to the target (e.g., a brown *rabbit*) or with the test item that was designed to look dissimilar from the target but belonged to the same category (e.g., a gray *squirrel*; but see Sloutsky and Fisher, 2004 for divergent arguments and data about the calibration of perceptual similarity in this study). The overall rate of category-match choices was above chance, both in preschool children and college students. These findings were taken as evidence that even young children hold a belief (or a naïve theory) that natural kind objects share a number of unobservable properties if they belong to the same category, and make inductive inferences on the basis of this belief. Subsequent studies reported similar findings in younger children and even infants (e.g., Gelman and Coley, 1990; Graham et al., 2004).

The similarity-based approach explains these findings through the contribution of the similarity of auditory features (i.e., linguistic labels in this case) to inductive inference. A mathematical model based on the SINC framework successfully captured the pattern of findings reported by Gelman and Markman (1986). Specifically, when visual features of the stimuli were ambiguous (e.g., the target matched one of the test objects on the shape dimension and the other test object on the texture and color dimensions; for details see Fisher, 2007), identical auditory features (such as linguistic labels) dramatically increased the perceptual similarity between pairs of objects (Sloutsky and Fisher, 2004, 2012). Thus, the same set of findings was interpreted in very different ways by the competing theoretical frameworks.

There has been considerable empirical support for both the naïve theory account (Gelman and Markman, 1986; Gelman and Coley, 1990; Gelman, 2003; Gelman and Davidson, 2013) and SINC (Sloutsky and Lo, 1999; Sloutsky and Fisher, 2004, 2012; Fisher and Sloutsky, 2005; Sloutsky et al., 2007; Badger and Shapiro, 2012). Nevertheless, the central claims of each account remain contested. Specifically, it is unclear (1) whether category-based induction is indeed a developmental default, and (2) whether linguistic labels promote inductive generalization by pointing to common categories or by increasing perceived similarity between objects denoted by identical (or phonologically similar) labels. The present research was designed to provide evidence related to both of these highly contested claims.

In Experiment 1, 3- to 5-year-old children completed an induction task with triads of objects in which visual similarity was in conflict with category membership, akin to the tasks used in much of the prior research (e.g., Gelman and Markman, 1986; Sloutsky and Fisher, 2004; Badger and Shapiro, 2012). However, because identical linguistic labels introduce the problem of interpretation, in Experiment 1 we did not use labels to communicate category membership. Instead, we selected items that are highly familiar to young children and whose category membership can be readily identified by young children *without labels being provided by an experimenter*. In other words, in contrast to the Gelman and Markman (1986) study, we did not use perceptually ambiguous items that required linguistic labels to disambiguate category membership. Using highly detailed and readily identifiable images removes the possibility that children may make inferences that are label-based but not category-based. The extant accounts make divergent predictions regarding children's performance patterns in this task: According to the naïve theory approach, children should make category-based inferences at above chance level even in the absence of linguistic labels, because labels are simply one way to point to object kind information (e.g., Gelman and Davidson, 2013). In contrast, SINC predicts that young children should make inferences on the basis of perceptual similarity, although it's possible that category-based inference may emerge in the course of development.

In Experiment 2, we asked children to make inductive inferences using the same triads as in Experiment 1, however in contrast to Experiment 1, each object was denoted by a linguistic label. In order to examine whether labels contribute to induction by pointing to object kind information or by increasing perceived similarity of presented entities, we used two different types of labels: Category Labels and Descriptor Labels. In the Category Labels condition, linguistic labels denoted object kind (e.g., *bird*, *clock*, etc.); but in the Descriptor Labels condition, linguistic labels described a salient property of the stimulus but did not provide information about object kind (e.g., *brown*, *round*, etc.). If labels promote inductive inference by pointing to categories, children should be likely to make label-based inferences in the Category Labels condition but not in the Descriptor Labels condition. However, if early in development labels promote induction by increasing perceived similarity among compared entities, children should make label-based inferences in both the Category Labels and the Descriptor Labels condition.

Finally, in both Experiments reported in this paper we asked children to make inductive inferences about animals as well as artifacts. The naïve theory approach suggests that natural kinds should promote inductive inferences to a greater degree than artifacts across all age groups tested in the reported studies (e.g., Gelman and Markman, 1986, p. 185; Gelman, 2003). In contrast, SINC makes no such prediction and suggests instead that inferences should be similarity-based for both types of items.

## Experiment 1

### Methods

#### Participants

Participants were 57 children: 18 five-year-olds (*M_age_* = 5.46 years, *SD* = 0.34 years, 9 females, 9 males), 21 four-year-olds (*M_age_* = 4.44 years, *SD* = 0.26 years, 10 females, 11 males), and 18 three-year-olds (*M_age_* = 3.66 years, *SD* = 0.27 years, 10 females, 8 males). Participants were recruited from local schools, preschools, and the Phipps Conservatory in Pittsburgh, Pennsylvania. Children were tested individually by trained research assistants. This experiment was carried out in accordance with the recommendations of the Carnegie Mellon University Institutional Review Board. Parental consent was obtained and the children provided verbal assent prior to participating in the study.

#### Design and procedure

##### Visual stimuli

**Figure 1:**
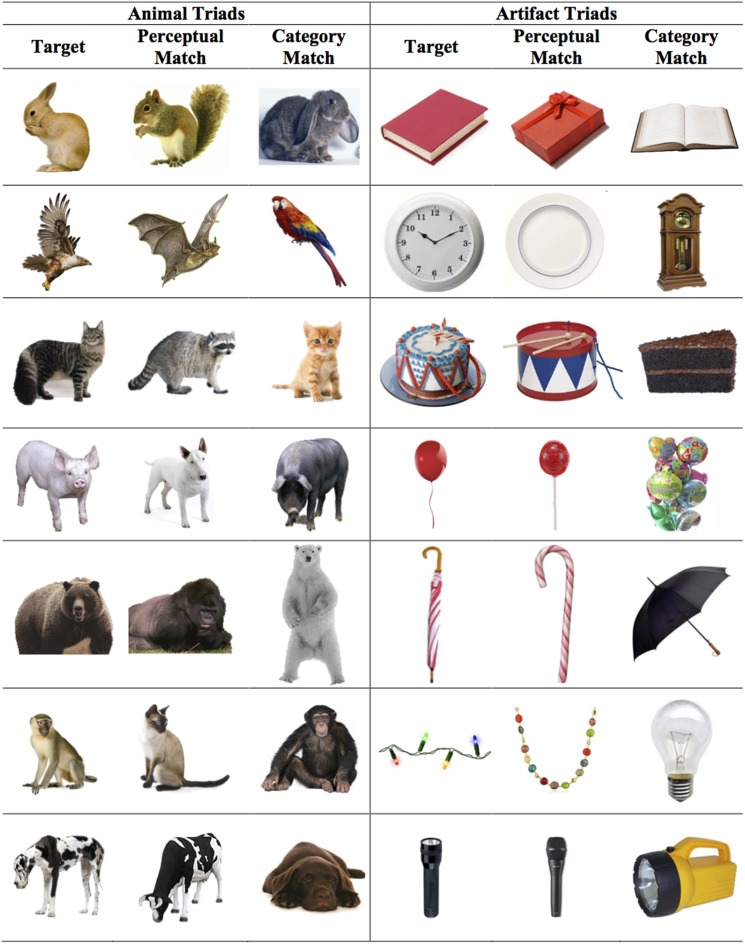
**Visual stimuli used in the Property Induction Task**. No labels were presented during the task. Each triad included a target, a category-match test item, and a perceptual-match test item. During the experiment, the target items were presented in the middle of the screen above the test items and approximately equidistant from each test item.

The visual stimuli included 14 triads displayed on a computer screen: 7 triads depicted artifacts and the remaining 7 triads depicted animals (see Figure 1). The stimuli were color photographs or detailed color pictures of objects. All triads consisted of a target item, category-match, and a perceptual-match. The triads were designed such that category membership was in conflict with perceptual similarity. Specifically, perceptual-match items matched the target item in both shape and color, and category-match items were selected such that they did not match the target item in either shape or color. Item selection was based on the Familiarity Calibration (described below), which ensured that children of this age group could readily identify the category membership of the items using common basic level labels.

##### Familiarity calibration

A separate group of preschool children (*N* = 13, *M_age_* = 4.61 years, *SD* = 0.20) participated in the calibration. The calibration consisted of a basic naming task: Participants were presented with a series of pictures displayed individually on a computer screen and asked to identify the object in the picture. If a child provided a subordinate level label, the experimenter was instructed to administer a prompt. For example, if children called the bird a “parrot” the experimenter would ask the child, *“What kind of animal is a parrot?”* If a child provided a semantically-similar label, the experimenter was instructed to administer a prompt. For example, if children called the monkey a “gorilla” the experimenter would ask the child what else the item might be called. If on any trial a child reported that they did not know the name of the test item, the experimenter was instructed to prompt the child to take their best guess.

Scoring of the calibration data was based on the following criteria: For perceptual-match items a correct response was classified as a response in which children provided the desired label or a label that was semantically-similar to the desired label (e.g., *necklace*-*beads*). Alternative labels were accepted as correct responses since the same pattern of category-based induction should be observed regardless of whether children use the intended label or an alternative label (i.e., children should make an inference from a bunny to another bunny regardless of whether they call the perceptual-match a squirrel or a chipmunk). A more stringent naming criteria was used for the target and category-match items compared to the perceptual-match items in order to ensure that children understood these items were the same kind of thing (a prerequisite for category-based reasoning) and thus ruling out the possibility that children are failing to engage in category-based reasoning due to lack of familiarity with the items. For target items and category-match items, a correct response was recorded if children provided the basic-level label. Correct responses also included occasions in which a child said an object name that included the basic-level label as part of his or her response (e.g., *“polar-bear”* for “*bear,”* or *“chocolate cake”* for *“cake”*). Subordinate labels or semantically-similar labels were accepted as correct responses only if children used the same label for both the target and the category-match (e.g., for the *bird* trials a child would receive credit if he or she labeled *both* the target and category-match as *“parrot”* because the same pattern of category-based induction should be observed since in both cases the child is identifying both items as the same kind of thing). See Table 1 for alternate labels that were accepted as correct responses for each trial.

**Table 1:** **Accuracy data for the Familiarity Calibration and examples of alternate labels scored as correct for each trial based on the aforementioned scoring criteria**.

**Test item**	**Proportion correct**	**Alternate labels**	**Test item**	**Proportion correct**	**Alternate labels**
Bunny	Target 1.00	Bunny-Rabbit	Book	Target 1.00	
	Category 1.00	Rabbit		Category 1.00	
Squirrel	Perceptual 0.92	Chipmunk^*^	Present	Perceptual 1.00	Gift
Bird	Target 0.69		Clock	Target 1.00	
	Category 0.77			Category 0.92	
Bat	Perceptual 1.00		Plate	Perceptual 0.77	Dish
					Bowl
Cat	Target 1.00	Kitty	Light	Target 0.92	Lights
	Category 1.00	Kitten		Category 1.00	Light bulb
		Kitty-cat			Christmas lights
Raccoon	Perceptual 0.85		Necklace	Perceptual 1.00	Jewelry
					Beads
					Bracelet
Pig	Target 1.00	Piggy	Cake	Target 1.00	Birthday cake
	Category 0.92	Piglet		Category 0.92	Cupcake
					Chocolate cake
Dog	Perceptual 0.92	Doggie	Drum	Perceptual 1.00	
		Puppy			
		Puppy-Dog			
Bear	Target 1.00	Polar-bear	Balloon	Target 1.00	
	Category 0.92	Teddy bear		Category 1.00	
Gorilla	Perceptual 0.85	Monkey	Lollipop	Perceptual 0.92	Sucker
		Chimpanzee			Candy
		Ape			
Monkey	Target 0.92		Umbrella	Target 0.85	
	Category 0.54			Category 1.00	
Cat	Perceptual 0.92	Kitty	Candy cane	Perceptual 0.92	Candy
		Kitten			Sucker
		Kitty-cat			
Dog	Target 1.00	Doggie	Flashlight	Target 1.00	Light
	Category 1.00	Puppy		Category 0.92	Torch
		Puppy-Dog			
Cow	Perceptual 1.00	Bull	Microphone	Perceptual 0.85	

* Although “chipmunk” was counted as a correct response, this response was not very common. Only 2 of the 13 (15%) children who participated in the Familiarity Calibration produced the label “chipmunk” for “squirrel.”

Mean accuracy for labeling the objects was high (*M* = 0.93, *SD* = 0.04). Children's high accuracy on the calibration suggests that the stimuli chosen for this study were highly familiar to preschool-age children, to the point that children could identify the objects correctly without an experimenter supplying category labels; see Table 1 for accuracy rates on individual test trials. Analysis of children's errors indicated that incorrect responses frequently occurred for trials in which the child generated an accurate subordinate level label but was unable to spontaneously generate the label at the basic category level. For example, children's accuracy on the *bird* category-match was 77%. However, of the 3 children who did not receive credit for this item, 2 of the 3 children provided an accurate level subordinate level label (e.g., “*parrot”*) while the remaining child indicated that they did not know the answer. Thus, the accuracy data presented here are a conservative estimate of children's familiarity with the test items and are more likely to underestimate than to overestimate children's knowledge. A Naming Task (described below), identical to the familiarity calibration, was administered after the experiment proper. The Naming Task served to ensure that children who participated in the study were familiar with the stimuli.

##### Property induction task

In the Property Induction Task children were presented with 14 triads. Each triad included a target, category-match, and perceptual-match (see Figure 1). The category-match and perceptual-match items were presented below the target item and approximately equidistant from the target. Category membership was communicated solely through detailed color photographs and no labels were used (cf. Smith and Heise, 1992). On every trial children were told that the target object had a particular property. All properties were one-syllable blank predicates chosen from the NOUN database (e.g., *fisp*, *wilp*, etc.; Horst, 2009). Then, the children were asked to generalize the target property to one of the test items (i.e., the category-match or the perceptual-match).

For the animal triads, children were told that the target item possessed an internal pseudo-biological property (e.g., *“This one has fisp cells inside”*) and asked to generalize the property to one of the test items. For the artifact triads, children were told what the target object was made of (e.g., *“This one is made of fupp”*) and asked to generalize the property to one of the test items. The screen location of the test items was counterbalanced and the trials were presented in one of two orders: In Order 1 the trials were randomized; for Order 2 the presentation order was the reverse of Order 1. The presentation order (1 vs. 2) was counterbalanced across participants.

##### Naming task

After the Property Induction Task, all participants completed a Naming Task to ensure that participants were familiar with the stimuli. The Naming Task was identical to the procedure utilized in the Familiarity Calibration: Participants were presented with a series of 42 pictures displayed individually on a computer screen. Children were asked to identify the object in the picture. Two presentation orders were created. In Order 1 the items were pseudo randomized with the following constraints: for any given triad, the target, category-match, and perceptual-match could not occur on successive trials. For Order 2 the items were administered in reverse order. The presentation order (1 vs. 2) was counterbalanced across participants.

### Results

The mean proportion of participants' choices of category-match items by age group and trial type are displayed in Table 2. Children's induction scores were submitted to a mixed ANOVA with age (5-, 4-, and 3-year-olds) as the between-subject factor and trial type (Animals, Artifacts) as the within-subject factor. The effect of trial type was not significant, *F*_(1, 54)_ = 0.84, *p* = 0.36. The interaction between trial type and age was also not significant, *F*_(2, 54)_ = 0.49, *p* = 0.62.

**Table 2:** **Mean proportions of choices of category-match items (*SD*) by age group and trial type**.

**Age Group**	**Animals**	**Artifacts**
5-Year-Olds	0.81 (0.22)	0.80 (0.22)
4-Year-Olds	0.61 (0.25)	0.61 (0.26)
3-Year-Olds	0.49 (0.24)	0.41 (0.21)

**Figure 2:**
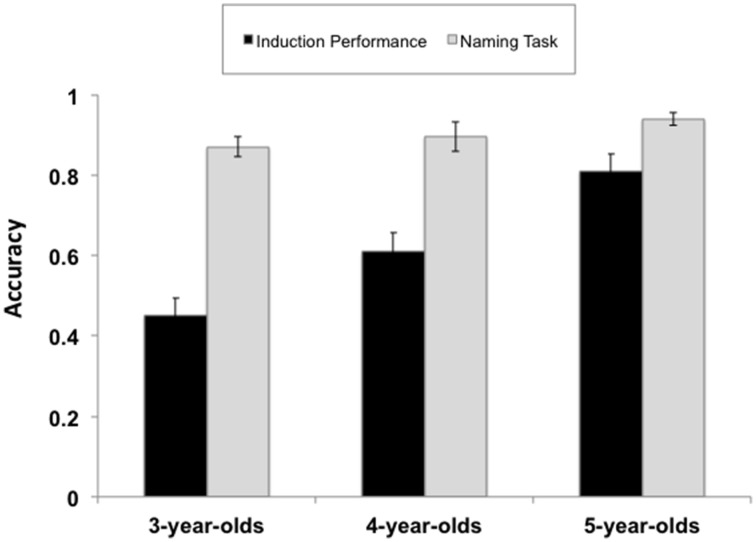
**Summary of children's performance on the Property Induction Task and the Naming Task across age groups**. Error bars represent the standard errors of the mean.

A significant effect of age was found, *F*_(2, 54)_ = 14.68, *p* < 0.0001, partial η^2^ = 0.35. *Post-hoc* Tukey tests indicated the following pattern of findings in children's proportion of category-match choices: 5-year-old children > 4-year-old children > 3-year-old children, all *p*s < 0.05. The effect size for the difference in induction performance between the 5-year-olds and the preschool children was large (5-year-olds vs. 4-year-olds Cohen's *d* = 1.0; 5-year-olds vs. 3-year-olds Cohen's *d* = 2.00), as was the effect size for the difference between 4- and 3-year-olds (Cohen's *d* = 0.80). Additionally, 4- and 5-year-old children selected category-match items at above chance (0.50) level, both one-sample *t*s ≥ 2.32, *p* < 0.05. However, the performance of 3-year-old children did not differ from chance, one-sample *t*_(17)_ = 1.10, *p* = 0.29 (see Figure 2).

Preschool children's difficulty on the Property Induction Task was clearly not due to lack of familiarity with the stimuli as evidenced by children's ability to label the stimuli with high accuracy (see Figure 2). Performance on the Naming Task^1^ was high across all three age groups (*M*_3−*year*−*olds*_ = 0.87, *SD* = 0.10; *M*_4−*year*−*olds*_ = 0.90, *SD* = 0.17; *M*_5−*year*−*olds*_ = 0.94, *SD* = 0.07). Although 5-year-olds exhibited superior performance on the Naming task compared to 3-year-olds [*t*_(34)_ = −2.49, *p* = 0.018], there was no significant difference in 3- and 4-year-olds' Naming Task performance [*t*_(36)_ = −0.558, *p* = 0.581 *ns*]. Critically, there was no significant difference in the naming accuracy between 5- and 4-year-olds, independent-samples *t*_(36)_ = 1.10, *p* = 0.28, despite dramatic differences in their induction performance. A test of equivalence was also conducted using Weber and Popova's (2012) Independent-Samples Equivalence Procedure in order to ascertain whether naming performance in 5-year-olds and 4-year-olds was statistically equivalent. The minimum substantial effect (Δ = 0.5) was selected based on Cohen's (1988) guidelines for a medium effect. The equivalence test was marginally significant suggesting that naming performance was comparable across these two age groups; *t*_(36)_ = 1.10, *p* = 0.057. For mean triad level data see Supplementary Material Table 1.

Although children exhibited high levels of accuracy on the Naming Task, children misidentified some of the test items. This leaves open the possibility that differences in induction performance stem from differences in children's level of familiarity with the stimuli. However, this possibility seems unlikely given the conservative scoring approach utilized in the Naming Task discussed above. Furthermore, the correlation between children's induction performance and accuracy on the Naming Task was not statistically significant in preschool children (in 3- and 4-year-olds both *rs* ≤ 0.18, both *ps* ≥ 0.49) or in older children (*r* = 0.39, *p* = 0.11 in 5-year-old children). Nevertheless, to fully evaluate the possibility that misidentification of items on the Naming Task contributed to children's induction performance, we reanalyzed the induction data correcting for performance on the Naming Task. Being able to accurately identify the target and category-match is a critical prerequisite for children to engage in category-based induction. Thus, we retained induction scores only for triads in which the mean accuracy on the Naming Task for the target and category-match were 89% or above. The Naming Task requires children to produce a label for each pictured item. Consequently, the task is more difficult than recognition tasks (e.g., the Peabody Picture Vocabulary Test, Dunn and Dunn, 2007) in which children simply point to a target item labeled by the experimenter. Therefore, we deemed that 89% accuracy on the Naming Task demonstrates sufficiently high level of familiarity with the items by most children to support spontaneous category-based inductive inferences. As a result of the correction, three induction triads were removed from the analysis: *bird-bird-bat*, *monkey-monkey-cat*, and *light-light-necklace*. After correcting for accuracy on the Naming Task, the rate of choices of category-match items was 46% in 3-year-old children (compared with the uncorrected mean of 45%), 61.47% in 4-year-olds (compared with the uncorrected mean of 61%), and 81.31% in 5-year-olds (compared with the uncorrected mean of 81%). Children's corrected induction scores were again submitted to a mixed ANOVA with age (5-, 4-, and 3-year-olds) as the between-subject factor and trial type (Animals, Artifacts) as the within-subject factor. The effect of trial type was not significant, *F*_(1, 54)_ = 0.021, *p* = 0.887. The interaction between trial type and age was also not significant, *F*_(2, 54)_ = 1.068, *p* = 0.351. A significant effect of age was found, *F*_(2, 54)_ = 12.273, *p* < 0.0001, partial η^2^ = 0.31. *Post-hoc* Tukey tests indicated the following pattern of findings in children's proportion of category-match choices: 5-year-old children made significantly more category-match choices than both 4-year-olds and 3-year-olds (both *ps* < 0.05), 4-year-old children also made more category-match choices than 3-year-old children but this difference was only marginally significant, *p* = 0.09. Therefore, children's pattern of induction performance remained largely unchanged after correcting for misidentifications on the Naming Task.

The results of Experiment 1 are not fully consistent with either the naïve theory approach or SINC. Specifically, the naïve theory approach predicts above chance selection of category-match items by 2 ½ years of age (Gelman and Coley, 1990), particularly for natural kind items. In contrast to this prediction, we observed a gradual age-related increase in the proportion of category-match choices, such that performance of 4- and 5-year-old children, but not of 3-year-old children, was above chance. Also in contrast to the predictions of the naïve theory approach there was no effect of item type (i.e., animals vs. artifacts) on performance. However, the observed pattern of results was also not fully consistent with the predictions of SINC (Sloutsky and Fisher, 2004). SINC predicts a developmental increase in children's ability to make inferences to category-match items, which was observed in Experiment 1. However, SINC also predicts that early in development children's inferences should be based predominantly on perceptual similarity; yet, not even the youngest participants in Experiment 1 were likely to make perceptual-match choices over category-match choices. In the General Discussion section we return to these findings and consider them in the context of other related findings in the literature.

## Experiment 2

In Experiment 2 we examined the mechanisms by which labels promote induction by investigating the influence of different label types (category and descriptor labels) on induction performance. Identical category labels are inherently confounded as they point to category information while providing an additional feature match between the labeled items. Furthermore, it has been suggested that early in life the attentional weight of auditory features is greater than that of visual features (e.g., Sloutsky and Napolitano, 2003; Robinson and Sloutsky, 2013); consequently, labels might make a particularly strong contribution to induction not by denoting object kind but by increasing perceived similarity of the labeled items (Sloutsky and Fisher, 2004, 2012). However, descriptor labels (i.e., labels that describe a perceptual characteristic of the item that is orthogonal to its category membership) only provide perceptual information and cannot be construed as a category marker. Therefore, in Experiment 2 we provided children with identical labels for the target and one of the test items (i.e., the category-match or perceptual-match) based on condition: In the Category Label condition we provided identical labels for the target and category-match (e.g., *bird*), and in the Descriptor Labels condition we provided identical labels for the target and the perceptual-match (e.g., *brown*).

By comparing children's induction performance with category and descriptor labels to a no-label baseline we can examine the mechanism by which labels promote induction. If labels promote induction by communicating object kind, the proportion of category-match choices should be above the No-Label Baseline in the Category Labels condition, but it should remain unchanged in the Descriptor Labels condition. If instead labels promote induction by increasing perceived similarity between the labeled entities, the proportion of category-match choices should be above the No-Label Baseline in the Category Labels condition and below the No-Label Baseline in the Descriptor Labels condition. In other words, both theoretical approaches make similar predictions for performance in the Category Labels condition; however, only SINC predicts that category labels *and* descriptor labels should affect children's induction performance.

### Methods

#### Participants

In this study, 160 children participated. Participants included: 5-year-olds (*N* = 53, *M_age_* = 5.24 years, *SD* = 0.24, 25 Males, 28 Females); 4-year-olds *N* = 54, *M_age_* = 4.53, *SD* = 0.23, 31 Males, 23 Females); and 3-year-olds (*N* = 53, *M_age_* = 3.65, *SD* = 0.22, 23 Males, 30 Females). Participants were recruited from local schools, preschools, and the Phipps Conservatory in Pittsburgh, Pennsylvania. Children were tested individually by trained research assistants. None of the children from Experiment 1 participated in Experiment 2. This experiment was carried out in accordance with the recommendations of the Carnegie Mellon University Institutional Review Board. Parental consent was obtained and the children provided verbal assent prior to participating in the study.

#### Design and procedure

##### Visual stimuli

Visual stimuli were identical to those used in the Property Induction Task in Experiment 1. The visual stimuli included 14 triads displayed on a computer screen: 7 triads referred to artifacts and 7 triads referred to animals.

##### Property induction task

The basic structure of the Property Induction Task was identical to Experiment 1: Children were presented with 14 triads; each triad included a target, a category-match, and a perceptual-match item (see Figure 1). However, in contrast to Experiment 1 in which no labels were used, in Experiment 2 children were assigned to one of three labeling conditions: No-Label Baseline, Category Labels, and Descriptor Labels). Thus, the label condition was a between-subjects factor.

On each trial, children were told that the target object had a particular property. All properties were one-syllable blank predicates chosen from the NOUN database (e.g., *fisp*, *wilp*, etc.; Horst, 2009). Twenty-eight blank predicates were selected: 14 blank predicates were randomly assigned to the No Label Condition and the remaining 14 were assigned to the Category Label and Descriptor Label conditions. The children were asked to generalize the target property to one of the test items (i.e., the category-match or the perceptual-match). In the No Label Condition the procedure was identical to Experiment 1. Children were told that the target object had a particular property (e.g., *“This one has lorp cells inside”*) and asked to generalize the property to either the category-match or the perceptual-match (e.g., “*Which of these* [category/perceptual matches] *do you think has lorp cells inside like this one* [target]*—this one or this one?* [category/perceptual matches]*”*). In the Category Labels Condition, the procedure was identical except children were told the basic level label for each item (e.g., *The* ***dog*** *has zerb cells inside*. *Which of these* [category/perceptual matches] *do you think has zerb cells inside like this dog* [target]*—the* ***dog*** *or the* ***cow****?* [category/perceptual matches]*”*). Similarly, in the Descriptor Labels Condition children were given a label that described a physical attribute of the object such as its color or shape (e.g., *The* ***spotted one*** *has zerb cells inside*. *Which of these* [category/perceptual matches] *do you think has zerb cells inside like this spotted one* [target]*—the* ***brown one*** *or the* ***spotted one****?* [category/perceptual matches]*”*). A full list of the linguistic stimuli utilized in the experiment is provided in Table 3. The screen location of the test items was counterbalanced and the trials were presented in one of two orders: In Order 1 the trials were randomized. For Order 2 the presentation order was simply reversed. Presentation order (1 vs. 2) was counterbalanced across participants.

**Table 3:** **Linguistic stimuli for Experiment 2**.

**Number**	**Category label condition**			**Descriptor label condition**			**No label condition**	**Properties Category and Descriptor labels conditions**	**Properties no label condition**
	**Target**	**Category match**	**Perceptual match**	**Target**	**Category match**	**Perceptual match**	**Target/Category/Perceptual match**		
1	Clock	Clock	Plate	Circle one	Long one	Circle one	This one	*bink*	*sarn*
2	Bird	Bird	Bat	Brown one	Colorful one	Brown one	This one	*wolp*	*fisp*
3	Flashlight	Flashlight	Microphone	Thin one	Thick one	Thin one	This one	*blap*	*husp*
4	Monkey	Monkey	Cat	Light colored one	Dark colored one	Light colored one	This one	*shill*	*nare*
5	Light	Light	Necklace	Little one	Big one	Little one	This one	*cusk*	*wilp*
6	Dog	Dog	Cow	Spotted one	Brown one	Spotted one	This one	*zerb*	*lorp*
7	Balloon	Balloon	Lollipop	Red one	Colorful one	Red one	This one	*glark*	*darg*
8	Pig	Pig	Dog	Small one	Large one	Small one	This one	*jate*	*tife*
9	Book	Book	Present	Closed one	Open one	Closed one	This one	*ratch*	*kern*
10	Cat	Cat	Raccoon	Gray one	Orange one	Gray one	This one	*lort*	*blick*
11	Umbrella	Umbrella	Candy Cane	Striped one	Black one	Striped one	This one	*nilt*	*pisk*
12	Bunny	Bunny	Squirrel	Tan one	Gray one	Tan one	This one	*vab*	*gree*
13	Cake	Cake	Drum	Round one	Straight one	Round one	This one	*gip*	*fupp*
14	Bear	Bear	Gorilla	Brown one	White one	Brown one	This one	*culp*	*terb*

##### Naming task

After the experiment proper, participants completed the Naming Task. The procedure for the Naming Task was identical to that in Experiment 1. Due to policy restrictions regarding how long children could be absent from their classroom, some children completed the Naming Task in a separate testing session. The average delay between the Induction Task and the Naming Task was 0.44 days (*SD* = 1.46 days, range: 0–8 days). The Naming Task served to ensure that the children in Experiment 2 were familiar with all of the stimuli.

### Results

Mean induction scores by age group, trial type, and condition are displayed in Table 4. Children's induction scores were submitted to a mixed ANOVA with age (3-, 4-, and 5-year-olds) and label condition (No Labels, Category Labels, and Descriptor Labels) as the between-subject factors and trial type (Animals, Artifacts) as the within-subject factor. The results indicated a significant effect of label condition [*F*_(2, 151)_ = 40.53, *p* = < 0.0001, partial η^2^ = 0.35] and a marginally significant effect of age [*F*_(2, 151)_ = 2.41, *p* = 0.09, partial η^2^ = 0.03]; the interaction between age and label condition was not significant, [*F*_(4, 151)_ = 1.93, *p* = 0.109, partial η^2^ = 0.05]. In contrast to Experiment 1, the effect of trial type was significant, *F*_(1, 151)_ = 7.81, *p* = 0.006, partial η^2^ = 0.05 with children obtaining on average higher induction scores for animals (*M* = 0.59, *SD* = 0.30) than for artifacts (*M* = 0.53, *SD* = 0.30). The interaction between trial type and age was not significant [*F*_(2, 151)_ = 1.11, *p* = 0.33]; nor was there a significant interaction between trial type and label condition [*F*_(2, 151)_ = 0.10, *p* = 0.90]. One the one hand, the effect of trial type in Experiment 2 is consistent with the predictions of the naïve theory approach and inconsistent with the predictions of SINC. On the other hand, this effect is somewhat difficult to interpret given the data shown in Table 4. For example, 3-year-olds made numerically more category-match choices for animal than for artifact trials only in the Descriptor Labels condition and in 4-year-old's this pattern was seen only in the Category Labels condition. In contrast, for 5-year-old's this pattern emerged in all labeling conditions. However these numerical differences in induction performance between animal and artifact triads were not consistently statistically significant: In 5-year-olds, there was no statistically significant difference for the Category Labels condition [*t*_(17)_ = 1.12, *p* = 0.27] and a marginally significant difference for the Descriptor Labels and No Labels conditions [both *ts* ≤ 2.05, *ps* ≥ 0.06].

**Table 4:** **Mean proportions of participants' choices of category-match items (*SD*) by age group, trial type, and label condition**.

**Age group**	**Condition**	**Animals**	**Artifacts**
5-Year-Olds	Descriptor labels	0.45 (0.32)	0.31 (0.27)
	Category labels	0.81 (0.19)	0.77 (0.20)
	No Labels (Baseline)	0.72 (0.29)	0.60 (0.26)
4-Year-Olds	Descriptor labels	0.31 (0.27)	0.33 (0.23)
	Category labels	0.85 (0.17)	0.75 (0.26)
	No Labels (Baseline)	0.56 (0.26)	0.54 (0.23)
3-Year-Olds	Descriptor labels	0.41 (0.20)	0.32 (0.24)
	Category labels	0.60 (0.30)	0.60 (0.36)
	No Labels (Baseline)	0.60 (0.23)	0.59 (0.20)

Next, in order to examine the mechanism by which labels promote induction we compared children's performance in each label condition (Category Labels and Descriptor Labels) to the No Label Baseline Condition. For this and all subsequent analyses, induction scores are collapsed across trial type (Animals and Artifacts).

#### Category labels vs. no labels baseline

**Figure 3:**
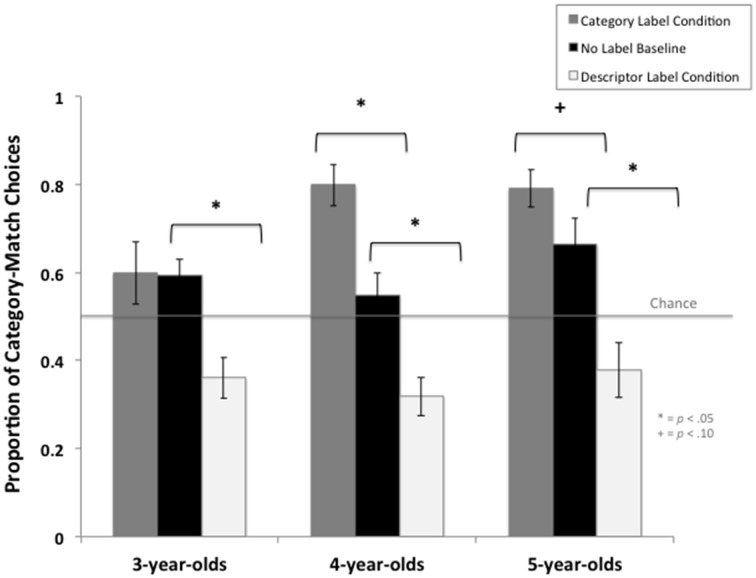
**Summary of children's performance on the Property Induction Task as a function of label condition (Category Label, No Label Baseline, and Descriptor Label conditions) across age groups**. Error bars represent the standard errors of the mean. Line indicates chance performance (0.50). ^*^p-value < 0.05;

The addition of category labels was not found to impact 3-year-old children's induction performance as there was no significant difference in 3-year-olds' propensity to select the category-match in the Category Label Condition (*M* = 0.60) compared to the No Label Baseline Condition (*M* = 0.59), independent sample *t*_(25.81)_ = 0.07, *p* = 0.94. A test of statistical equivalence was also conducted using Weber and Popova's (2012) Independent-Samples Equivalence Procedure in order to ascertain whether 3-year-olds' induction performance in the Category label and No Label Baseline conditions were statistically equivalent. The minimum substantial effect (Δ = 0.5) was selected based on Cohen's (1988) guidelines for a medium effect. The equivalence test was significant suggesting that 3-year-olds' induction performance was comparable across these two conditions; *t*_(33)_ = 0.07, *p* = 0.006. In contrast, providing category-labels did influence the performance of older children: 4-year old children selected the category-match items significantly more in the Category Label Condition (0.80) compared to the No Label Baseline Condition (0.55), independent sample *t*_(34)_ = 3.57, *p* = 0.001. This effect was large, Cohen's *d* = 1.19. In 5-year-old children the difference in induction performance between the Category Label (0.79) and No Label Baseline Condition (0.66) was marginally significant, independent sample *t*_(34)_ = 1.75, *p* = 0.09, Cohen's *d* = 0.60, see Figure 3. Furthermore, the addition of category labels did not support 3-year-old children's induction performance as they did not select the category-match above chance levels (0.50), single sample *t*_(17)_ = 1.39, *p* = 0.18. In contrast, when 4-year-old children were provided with the category-label they selected the category-match items at rates significantly above chance (0.50), single sample *t*_(17)_ = 6.35, *p* < 0.0001. Five-year-olds selected category-match items above chance levels (0.50) both in the Category Label Condition and No Label Baseline, both single sample *t*s > 2.80, both *p*s < 0.05.

In the Category label Condition, induction performance varied as a function of age. Older children (4- and 5-year-olds) were more likely to select category-match items than 3-year-olds, both independent sample *ts* ≥ 2.31, *ps* = 0.03. These effects were large (3-year-olds vs. 4-year-olds Cohen's *d* = 0.78, 3-year-olds vs. 5-year-olds Cohen's *d* = 0.77). At the same time, there was no difference in induction performance between 4- and 5-year old children, independent sample *t*_(34)_ = 0.12, *p* = 0.90. Weber and Popova's (2012) Independent-Samples Equivalence Procedure confirmed that 4- and 5-year-olds' induction performance in the Category Label Condition was comparable across these two age groups; Δ = 0.5, *t*_(34)_ = 0.12, *p* = 0.006.

It should be noted that results of the No Labels Baseline partially replicate the findings reported in Experiment 1. Specifically, 5-year-old children selected category-match items at above chance level even in the absence of category labels [*M* = 0.66, single-sample *t*_(17)_ = 2.81, *p* < 0.05], whereas 4-year-old children's performance was not different from chance in the No Label Baseline [*M* = 0.55, single sample *t*_(17)_ < 1, *ns*]. However, unlike Experiment 1, the performance of 3-year-old children was above chance in the No Label Baseline [*M* = 0.60, *t*_(16)_ = 2.46, *p* < 0.05] and not significantly different from the performance of older children (both single sample *t*s < 1.1, *p*s > 0.488). It is unclear why performance of 3-year-old children was different between Experiments 1 and 2 in the No Labels Baseline condition. However, in light of 3-year-old's chance-level performance in the Category Labels condition of Experiment 2 [*M* = 0.60, *t*_(17)_ = 1.39, *p* > 0.18] and in light of chance-level performance of 4-year-old children in the No Labels Baseline (both in Experiments 1 and 2), it would be premature to conclude that young children consistently make category-based inferences. 3-year-olds' above-chance performance in the No Labels Baseline in Experiment 2 appears to be an anomaly to the overall pattern.

#### Descriptor labels vs. no labels baseline

In contrast to performance in the Category Labels Condition, the addition of descriptor labels was found to significantly impact children's induction performance in all three age groups: children were significantly *less* likely to select the category-match in the Descriptor Label Condition compared to the No Label Baseline Condition (all independent sample *ts* ≥ 3.34, all *ps* ≤ 0.002), and the effect sizes were large (3-year-olds Cohen's *d* = 1.27, 4-year-olds Cohen's *d* = 1.12, 5-year-olds Cohen's *d* = 1.12), see Figure 3.

The youngest children (3- and 4-year-olds) selected category-match items below chance levels (0.50), both single sample *ts* ≥ 3.03, *ps* ≤ 0.008. Five-year-old children selected the category-match at rates marginally below chance level, single sample *t*_(16)_ = 1.98, *p* < 0.065. Furthermore, there was no significant difference in induction performance across all three age groups (*M*_3−*year*−*olds*_ = 0.36, *M*_4−*year*−*olds*_ = 0.32, *M*_5−*year*−*olds*_ = 0.38), all independent sample *ts* ≤ 0.78, *ps* ≥ 0.44. Weber and Popova's (2012) Independent-Samples Equivalence Procedure confirmed that induction performance in the Descriptor Label Condition was comparable across all three age groups: Δ = 0.5, all *ts* = 0.22 all *ps* = 0.036.

Similar to the results of Experiment 1, children exhibited high levels of performance in the Naming Task^2^. Performance on the Naming Task was high in all three age groups (*M*_3−*year*−*olds*_ = 0.88, *SD* = 0.10; *M*_4−*year*−*olds*_ = 0.94, *SD* = 0.06; *M*_5−*year*−*olds*_ = 0.96, *SD* = 0.05) indicating that children were highly familiar with the objects used in the present study and suggesting that the children possessed the prerequisite knowledge to perform category-based induction. Although children generally exhibited a high level of accuracy on the Naming Task, 4- and 5-year-old children obtained higher Naming Task scores than 3-year-olds (both independent sample *ts* ≥ −4.18, *ps* < 0.0001). There was no significant difference in the naming performance of 4- and 5-year-olds [*t*_(105)_ = −1.14, *p* = 0.26]. A test of equivalence was also conducted using Weber and Popova's (2012) Independent-Samples Equivalence Procedure in order to ascertain whether naming performance in 4- and 5-year-olds was statistically equivalent. The equivalence test was significant suggesting that naming performance was comparable across these two age groups; Δ = 0.5, *t*_(105)_ = −1.14, *p* < 0.0001. For mean triad level data see Supplementary Material Table 2.

The results show that in 4- and 5-year-old children both category and descriptor labels influence children's induction performance compared to the No Label Baseline, with category-labels increasing children's tendency to select the category-match and descriptor labels increasing children's tendency to select the perceptual-match. In 4-year-old children, the effect of descriptor and category labels on induction performance was of comparable magnitude (i.e., Cohen's *d* = 1.12 and 1.19, respectively). In 5-year-old children, the effect size of descriptor labels was nearly double the effect size of category labels (Cohen's *d* = 1.12 and 0.60, respectively). In contrast, for 3-year-olds only descriptor labels had a large effect on children's performance (Cohen's *d* = 1.27), whereas category labels had no effect. The observed pattern of results is in line with predictions from SINC and conflict with predictions from the naïve theory. Specifically, the results suggest that a large part of the effect of labels on induction stems from identical labels increasing the overall perceived similarity of objects.

## Discussion

The experiments presented here were designed to contrast the predictions of the naïve theory approach (Gelman, 2003) and SINC (Sloutsky and Fisher, 2004, 2012) with regards to two highly contentious issues: (1) whether children engage in category-based induction overlooking conflicting appearances from early in development, and (2) whether linguistic labels promote induction by pointing to categories or by increasing the overall perceived similarity of presented items. In Experiment 1 we asked 3- to 5-year-old children to make inductive inferences about highly familiar objects (both animals and artifacts), with detailed images obviating the need for linguistic labels to disambiguate category membership. In other words, Experiment 1 avoided confounding category-based and label-based induction that was problematic in some of the previous studies. Results of Experiment 1 were partially consistent with both the naïve theory and SINC. As predicted by SINC and in contrast to the predictions of the naïve theory, 3-year-old children did not make inferences to category-match items when a perceptual-match was available, even though the categories used in the task were highly familiar to children. Also consistent with the predictions of SINC, there was a gradual increase between three and five years of age in children's tendency to make inferences to category-match items over perceptual-match items, although the timing of this transition was considerably accelerated compared to prior studies which suggest that this transition does not happen until about seven to nine years of age (Sloutsky and Spino, 2004; Badger and Shapiro, 2012). The findings of Experiment 2 were mostly consistent with this pattern, with the exception of 3-year-old children making inferences to category match items at above chance level in the absence of category labels (but interestingly, not when category labels were provided, suggesting that this is not a robust effect). However, in contrast to the predictions of SINC but consistent with the predictions of the naïve theory, children were not limited to making inferences *solely* on the basis of perceptual similarity: no age group made systematic inferences to perpetual-match items when appearances conflicted with category membership.

Experiment 2 examined whether linguistic labels affect children's inductive inferences by communicating object kind (as predicted by the naïve theory) or by increasing the perceived similarity of the objects denoted by identical labels (as predicted by SINC). Recall that both theoretical approaches predict that Category Labels should affect children's induction compared to the No-Labels Baseline Condition; however, only SINC predicts that Category and Descriptor Labels should affects children's performance. Consistent with the predictions of SINC, and in contrast to the predictions of the naïve theory approach, induction performance in 4- to 5-year-old children was affected by both types of labels, such that Category Labels increased the proportion of category-match responses and Descriptor Labels increased the proportion of perceptual-match items compared to the No Labels Baseline Condition. In younger children, only the Descriptor but not Category Labels affected children's induction performance. Therefore, identical labels affected younger children's performance only when these labels aligned with perceptual similarity but not with common category membership, a finding that again can be explained in a straightforward manner by SINC but is problematic for the naïve theory.

There was an inconsistent pattern of findings with regards to the ontological status of the objects. There were no differences in the pattern of inferences with animals and artifacts for any of the three age groups in Experiment 1, but in Experiment 2 children were more likely to make inferences to category-match items with animals than with artifacts. This pattern in Experiment 2 became more robust with age: 3-year-old children showed this pattern only in the Descriptor Labels condition, 4-year-old children only in the Category Labels condition, and 5-year-old children in all three label conditions (i.e., No Labels Baseline, Category Labels, and Descriptor Labels conditions). In this regard, the findings of Experiment 2 are consistent with the findings recently reported by Fisher et al. (2015a, Experiment 2). In that study, children were asked to make inductive inferences about familiar items that were described as “hiding” behind identical doors in the beginning of their second year in preschool (*M_age_* = 4.33 years) and then at the end of the school year (*M_age_* = 4.77). In the beginning of the school year, there were no differences in choices of category-match items from different ontological groups (i.e., animals, inanimate natural kinds, and artifacts), but at the end of the school year children became more likely to make inferences to category-match items for animals and inanimate natural kinds than for artifacts. These findings, taken together with the findings in Experiment 2 in the present study, suggest that in the course of development, children learn that different ontological classes support inductive inferences to a different degree, rather than having this assumption as an early bias that is independent of experience (cf. Gelman, 2003).

### Implications for developmental theories of induction

Overall, it appears that several aspects of the reported findings are better explained by SINC than the naïve theory. At the same time, the lack of tendency to choose perceptual-match items even among the youngest participants is problematic for SINC. Additionally, SINC has trouble accounting for the findings of prior studies in which familiar objects were described as “hiding” behind identical doors (such that children could not rely on the overlap in visual features to make inferences) and category information was communicated by semantically-similar labels (such that children could not rely on the overlap in auditory features to make inferences) (Fisher et al., 2011; Godwin et al., 2013; Fisher et al., 2015b; cf. Gelman and Markman, 1986). Under these task conditions, most 4-year-old children performed at chance level: when told about an unobservable property of a “sheep,” they were equally likely to make an inference to a “lamb” (category-match) and a “cow” (lure). However, over half of 5-year-olds and nearly all 6-year-olds made inferences to the category-match items. These findings challenge the predictions of SINC that (1) young children make inferences solely on the basis of perceptual similarity, and that (2) labels contribute to induction in children solely through featural overlap.

Although the naïve theory and SINC are the two dominant accounts of induction early in development, some researchers have advocated the idea that domain knowledge (vs. an abstract “initial bias” toward category-based induction) influences the types of inductive inferences that children make. For example, Chi et al. (1989) identified 6-year-old children who had substantial prior knowledge about dinosaurs (i.e., dinosaur experts) and children who possessed little knowledge about dinosaurs. Children who were dinosaur experts tended to make category-based inferences about dinosaurs (e.g., “he is probably a good swimmer… cause duckbills are good swimmers”; p. 48). In contrast, children who knew little about dinosaurs, tended to make inferences that were based on the appearance of the stimuli (e.g., “could walk real fast cause he has giant legs,” p. 49). Similarly, Inagaki (1990) reported that urban children who had experience caring for a goldfish showed a distinct pattern of inductive inferences compared to children who did not have a pet. Additionally, these children were able to use their acquired knowledge as a basis for reasoning about other aquatic animals.

The findings above taken together with the findings of Experiment 1 and the findings reported in several other studies (Fisher et al., 2011; Long et al., 2012; Godwin et al., 2013) present a striking contrast to studies suggesting that children have difficulty making category-based inferences until about 9 years of age (Sloutsky and Spino, 2004; Sloutsky et al., 2007; Badger and Shapiro, 2012). One potentially important distinction between these two sets of studies is *familiarity* of the stimuli: the former studies used real familiar categories, whereas the latter used novel artificial categories. Thus, it appears that children do not have an abstract “initial bias” toward category-based induction as suggested by the naïve theory (Gelman, 2003; Gelman and Davidson, 2013); at the same time it appears that children base inferences primarily on perceptual features only when objects belong to newly-learned artificial categories. Therefore, we suggest a revised version of the similarity-based account, which we briefly describe below.

#### The perceptual and representational similarity (PaRS) account of inductive generalization

Fisher et al. (2015a) recently proposed a revised version of the similarity-based account of inductive generalization. The basic premise of this account is that one can distinguish two forms of featural similarity: perceptual and representational similarity. Perceptual similarity refers to features that can be compared on-line and in-the-moment, and representational similarity refers to the featural overlap in mental representations. Within this proposal (to which we will refer to as PaRS—for Perceptual and Representational Similarity), representational similarity refers to the knowledge acquired through experience (e.g., knowledge of what things are called, how things look, how they may be used, etc.) rather than the knowledge that one may have independent of experience, such as essentialist beliefs (Gelman, 2003). According to PaRS, both types of similarity contribute to inductive generalization, and the probability of an inference is a function of the overall featural overlap among the presented objects (Fisher et al., 2015b; Fisher, 2015).

Understanding the mechanisms of developmental change in inductive reasoning with newly-learned categories remains an important question for future research. At the same time, PaRS (in contrast to the naïve theory and SINC) offers an account of developmental changes in induction with familiar categories. Specifically, PaRS suggests that developmental changes in induction with familiar categories stem from developmental changes in representational similarity. A number of computational studies suggest that early in development concepts are organized on the basis of a small number of salient features, leading to formation of poorly differentiated representations. However, as the number of features associated with a concept increases, representations become progressively more differentiated according to kind-based relations, because objects of similar kind share a greater number of features with each other than with objects of a different kind (Rogers and McClelland, 2004; Hills et al., 2009; Kemp and Tenenbaum, 2008). For example, both bats and birds can fly; when little else is known about bats and birds, their representations may be highly similar. However, as one learns that birds—but not bats—lay eggs, have beaks and feathers, and build nests, the representations of birds and bats should become more distinct.

Increase in semantic differentiation on the basis of an increase in the number of known features has been captured by different computational models of semantic development (for discussion see Fisher et al., 2015b). There is also recent empirical evidence that supports this idea (Unger et al., 2014, under review; Fisher et al., 2015b). Furthermore, there is evidence that individual differences in semantic differentiation of familiar animal concepts are related to children's ability to make category-consistent inferences. Specifically, semantic differentiation of familiar animal concepts was found to be positively related to the tendency to make category-consistent inferences both in a cross-sectional study of 4- to 7-year-old children (Fisher et al., 2015b) as well as in a longitudinal study of 4- to 5-year-old children (Fisher et al., 2015a). Furthermore, individual differences in semantic differentiation of animal concepts were a better predictor of children's performance on the inductive reasoning task than general intelligence: General intelligence was related to children's semantic differentiation, but only the latter made a direct (unmediated) contribution to induction performance (Fisher et al., 2015a).

### Mechanisms of the effect of linguistic labels on induction

Effect of linguistic labels on induction has been documented in a number of studies (e.g., Gelman and Markman, 1986; Sloutsky et al., 2001; Welder and Graham, 2001; Sloutsky and Fisher, 2004). At the same time, the mechanism of these effects has remained contested, with some researchers suggesting that identical labels contribute to induction by pointing to categories (e.g., Gelman and Markman, 1986; Gelman and Coley, 1991; Waxman and Gelman, 2009) and other researchers suggesting that labels are object features and contribute to induction by increasing overall perceived similarity of objects (Sloutsky and Fisher, 2004, 2012; Sloutsky, 2009; Deng and Sloutsky, 2012). In the Category Labels Condition of Experiment 2, labels could contribute to induction both through increasing overall perceptual similarity and through communicating category membership; however, in the Descriptor Labels Condition, labels could not possibly communicate category membership and could only contribute to induction by increasing overall perceived similarity. In both 4- and 5-year-old children, both descriptor labels and category labels had a comparable effect on induction. This suggests that a large part of the effect of labels on induction stems from identical labels increasing the overall perceived similarity of objects, rather than from labels communicating category membership. Interestingly, in 3-year-old children only descriptor labels but not category labels promoted inductive inferences. In other words, in the youngest children in this study, linguistic labels promoted induction only when they aligned with visual similarity but not with category membership, again suggesting that perceptual information plays a particularly important role in induction early in development.

Overall, the findings of Experiment 2 clearly support the predictions of SINC and pose a challenge for the naive theory approach. However, as discussed above, starting at approximately 5 years of age children begin to make category-consistent inferences relying on semantically-similar labels (e.g., *lamb-sheep*) in the absence of useful visual information to guide their induction (Fisher et al., 2011, 2015a; Godwin et al., 2013). These findings are difficult to reconcile with SINC as it is currently formulated (Sloutsky and Fisher, 2004, 2012). According to PaRS, labels are indeed object features (as suggested by SINC) and can increase perceived similarity of presented objects; however, labels are also features that can point to other features. For example, when one sees an outline of a bird, this visual feature can activate other features stored in memory, such as its associated label (e.g., *bird*), mode of locomotion (e.g., *can fly*) and so on. Similarly, when one hears a label (e.g., *bird*), this linguistic feature can activate other features stored in memory (such as *has feathers*, *can fly*, and *lays eggs*). Early in development, when few features are known and semantic representations are therefore poorly differentiated (e.g., Hills et al., 2009; Fisher et al., 2015b), the contribution of linguistic labels to induction might occur primarily via increasing overall perceived similarity of objects under consideration; however, as semantic representations become more differentiated in the course of development, familiar labels may begin to contribute to induction via not only increasing perceived similarity but also activating other object features in memory. The principal difference between this proposal and the proposal of the naïve theory approach is as follows: according to the naïve theory approach linguistic labels point to categories and are distinct from all other object features, whereas according to PaRS (as well as SINC) linguistic labels are considered to function in a manner similar to other object features. For example, according to PaRS and SINC (and in contrast to the naïve theory), category labels are not granted a special status compared to other types of auditory features (e.g., sounds produced by objects, such as animal sounds). Although there is considerable evidence that linguistic labels may *become* category markers by adulthood (Yamauchi and Markman, 1998, 2000; Yamauchi et al., 2007; Yamauchi and Yu, 2008), the results of Experiment 2 provide additional evidence that labels do not *start out* as such.

### Broader implications

Outside of the debate about the mechanisms of inductive generalization early in development, the results reported here are consistent with a broader body of research on conceptual development. Specifically, a number of studies suggest that detection and processing of category-level information show a marked improvement during the preschool and early school years. For example, in match-to-sample tasks participants are asked to identify a test item that best matches a target item. Typically, the target is semantically-similar to one test item, and thematically- or perceptually-similar to a non-category-match item (e.g., Smiley and Brown, 1979; Tversky, 1985; Deák et al., 2002; Fisher, 2011). Children's ability to make taxonomic matches in the presence of perceptual lures (e.g., *chocolate cake*—*slice of birthday cake*—*brown top-hat*) or thematic lures (e.g., *carrot*—*rabbit*—*tomato*) improves gradually between three and six years of age. Similar trends emerge in free sorting tasks in which children are asked to sort a number of items that can be cross-classified into multiple groups (Blaye et al., 2006; Unger et al., 2014). Specifically, 4-year-old children often produce idiosyncratic groupings or thematic groupings, whereas by 8 years of age children's thematic groupings become embedded within taxonomic groupings. Literature on the development of analogical reasoning has similarly documented that up to 5 years of age children often focus on perceptual commonalities when interpreting metaphors and reasoning by analogy, whereas older children and adults tend to focus on the so-called relational commonalities, such as shared function, causal roles, and category membership (e.g., Gentner, 1978, 1988; Rattermann and Gentner, 1998; Gentner and Smith, 2012).

Therefore, perceptual commonalities clearly influence children's performance on a broad range of cognitive tasks from early in development, whereas the influence of category-level information increases gradually with development and learning. At the same time, the influence of category knowledge can also be seen early in development under less demanding task conditions. For example, in the absence of perceptual and thematic lures, even 2- and 3-year-old children can select taxonomic matches in the match-to-sample tasks (Nguyen, 2007). The present findings contribute to the large body of research suggesting that the early grasp of taxonomic relations is clearly tenuous and not sufficiently robust when perceptual or thematic response options are available to children, but shows a marked improvement over the preschool years.

### Conflict of interest statement

The authors declare that the research was conducted in the absence of any commercial or financial relationships that could be construed as a potential conflict of interest.

## Footnotes

^1^One child did not provide a verbal response and thus did not contribute any Naming task data.

^2^ Due to technical difficulties, one child only completed 40 of the 42 Naming Task trials.
