# Supplementary material for: Inductive generalization with familiar categories: developmental changes in children's reliance on perceptual similarity and kind information
Source: Front Psychol. 2015 Jul 7;6:897. doi: 10.3389/fpsyg.2015.00897 (PMC4493371; doi:10.3389/fpsyg.2015.00897)
Supplement: Supplementary file 1 [file Table1.PDF]

Supplementary Table A

Mean triad level data by age group for the Naming Task and Induction performance in Experiment 1

		Naming Performance			Induction			
Type	Triads	3's	4's	5's	3's	4's	5's	Property
Target	Cake	0.89	0.95	1.00				
Category-Match	Cake	0.94	1.00	1.00	0.50	0.81	0.94	*fupp*
Perceptual-Match	Drum	0.94	0.90	0.94				
Target	Bunny	0.94	0.95	0.94				
Category-Match	Bunny	1.00	0.95	1.00	0.50	0.67	0.78	*gree*
Perceptual-Match	Squirrel	0.56	0.90	1.00				
Target	Balloon	0.94	0.95	1.00				
Category-Match	Balloon	1.00	0.95	1.00	0.28	0.67	0.83	*darg*
Perceptual-Match	Lollipop	0.67	0.80	0.83				
Target	Bird	0.78	0.90	0.94				
Category-Match	Bird	0.67	0.80	0.94	0.44	0.52	0.83	*fisp*
Perceptual-Match	Bat	0.83	0.90	0.89				
Target	Clock	0.89	0.90	1.00				
Category-Match	Clock	0.83	0.95	0.94	0.56	0.38	0.83	*sarn*
Perceptual-Match	Plate	0.89	0.70	0.94				
Target	Cat	1.00	1.00	0.94				
Category-Match	Cat	1.00	0.95	1.00	0.72	0.76	0.83	*blick*
Perceptual-Match	Raccoon	0.61	0.55	0.56				
Target	Flashlight	0.89	0.95	0.94				
Category-Match	Flashlight	0.94	0.90	0.94	0.50	0.43	0.72	*husp*
Perceptual-Match	Microphone	0.56	0.70	0.83				
Target	Pig	1.00	0.95	1.00				
Category-Match	Pig	0.89	0.95	1.00	0.17	0.57	0.78	*tife*
Perceptual-Match	Dog	0.94	1.00	1.00				
Target	Umbrella	0.94	0.95	1.00				
Category-Match	Umbrella	1.00	0.95	1.00	0.28	0.67	0.78	*pisk*
Perceptual-Match	Candy Cane	0.89	0.95	0.94				
Target	Dog	0.94	0.90	0.94				
Category-Match	Dog	1.00	1.00	1.00	0.61	0.24	0.72	*lorp*
Perceptual-Match	Cow	0.89	0.95	1.00				
Target	Monkey	1.00	0.85	1.00				
Category-Match	Monkey	0.83	0.75	0.89	0.44	0.81	0.83	*nare*
Perceptual-Match	Cat	0.83	0.90	0.94				
Target	Book	1.00	0.95	1.00				
Category-Match	Book	0.89	0.95	1.00	0.44	0.86	0.83	*kern*
Perceptual-Match	Present	0.83	0.80	0.72				
Target	Bear	0.94	0.95	1.00				
Category-Match	Bear	1.00	0.95	1.00	0.56	0.71	0.89	*terb*
Perceptual-Match	Gorilla	0.72	0.85	0.78				
Target	Light	0.78	0.90	0.94				
Category-Match	Light	0.61	0.80	0.89	0.33	0.43	0.67	*wilp*
Perceptual-Match	Necklace	0.83	0.85	0.94				
