# Supplementary material for: Inductive generalization with familiar categories: developmental changes in children's reliance on perceptual similarity and kind information
Source: Front Psychol. 2015 Jul 7;6:897. doi: 10.3389/fpsyg.2015.00897 (PMC4493371; doi:10.3389/fpsyg.2015.00897)
Supplement: Supplementary file 2 [file Table2.PDF]

Supplementary Table B

Mean triad level data by age group for the Naming Task and Induction performance in Experiment 2

		Naming Performance			Induction No Labels			Induction Category Labels			Induction Descriptor Labels		
**Type**	**Triads**	**3's**	**4's**	**5's**	**3's**	**4's**	**5's**	**3's**	**4's**	**5's**	**3's**	**4's**	**5's**
Target	Cake	0.92	0.98	1.00									
Category-Match	Cake	0.92	1.00	1.00	0.71	0.89	0.78	0.67	1.00	0.94	0.56	0.50	0.50
Perceptual-Match	Drum	0.94	1.00	1.00									
Target	Bunny	0.91	1.00	1.00									
Category-Match	Bunny	0.96	0.96	1.00	0.65	0.50	0.56	0.50	0.94	0.94	0.33	0.50	0.39
Perceptual-Match	Squirrel	0.89	0.89	0.91									
Target	Balloon	1.00	1.00	1.00									
Category-Match	Balloon	1.00	1.00	1.00	0.59	0.50	0.78	0.61	0.67	0.78	0.28	0.50	0.50
Perceptual-Match	Lollipop	0.85	0.91	0.98									
Target	Bird	0.74	0.85	0.81									
Category-Match	Bird	0.64	0.80	0.87	0.41	0.50	0.78	0.56	0.61	0.61	0.44	0.33	0.44
Perceptual-Match	Bat	0.83	0.96	0.98									
Target	Clock	0.98	0.96	0.98									
Category-Match	Clock	0.91	0.94	0.96	0.71	0.44	0.50	0.61	0.61	0.72	0.39	0.22	0.22
Perceptual-Match	Plate	0.74	0.85	0.96									
Target	Cat	1.00	1.00	1.00									
Category-Match	Cat	1.00	1.00	1.00	0.71	0.83	0.72	0.78	1.00	1.00	0.61	0.61	0.44
Perceptual-Match	Raccoon	0.60	0.69	0.83									
Target	Flashlight	0.87	0.89	0.98									
Category-Match	Flashlight	0.79	0.96	0.94	0.53	0.39	0.50	0.61	0.72	0.72	0.17	0.28	0.22
Perceptual-Match	Microphone	0.53	0.78	0.81									
Target	Pig	1.00	1.00	1.00									
Category-Match	Pig	0.96	0.96	0.96	0.65	0.61	0.78	0.56	0.94	0.83	0.28	0.22	0.56
Perceptual-Match	Dog	0.94	0.98	1.00									
Target	Umbrella	0.92	0.98	0.96									
Category-Match	Umbrella	0.94	0.98	1.00	0.59	0.50	0.56	0.50	0.50	0.61	0.11	0.28	0.28
Perceptual-Match	Candy Cane	0.72	0.98	0.98									
Target	Dog	0.87	0.93	0.98									
Category-Match	Dog	0.91	0.98	1.00	0.47	0.22	0.67	0.50	0.61	0.83	0.28	0.00	0.33
Perceptual-Match	Cow	0.94	0.96	0.94									

Target	Monkey	0.89	0.96	0.98									
Category-Match	Monkey	0.77	0.85	0.80	0.76	0.56	0.78	0.67	0.89	0.67	0.22	0.22	0.39
Perceptual-Match	Cat	0.92	0.94	0.98									
Target	Book	0.98	1.00	0.98									
Category-Match	Book	1.00	1.00	1.00	0.59	0.61	0.78	0.56	0.78	0.78	0.33	0.22	0.22
Perceptual-Match	Present	0.85	0.96	0.80									
Target	Bear	0.98	0.98	1.00									
Category-Match	Bear	0.96	0.98	0.98	0.53	0.67	0.78	0.67	0.94	0.78	0.67	0.28	0.50
Perceptual-Match	Gorilla	0.72	0.94	0.89									
Target	Light	0.89	0.96	0.93									
Category-Match	Light	0.81	0.93	0.96	0.41	0.44	0.33	0.61	0.94	0.83	0.39	0.28	0.11
Perceptual-Match	Necklace	0.85	0.94	0.96									
